# HSPA8 Is a New Biomarker of Triple Negative Breast Cancer Related to Prognosis and Immune Infiltration

**DOI:** 10.1155/2022/8446857

**Published:** 2022-11-21

**Authors:** Bicheng Ying, Wenting Xu, Yan Nie, Yongtao Li

**Affiliations:** ^1^Department of Breast Surgery, Affiliated Cancer Hospital of Xinjiang Medical University, Urumqi, China; ^2^Yanqing District Hospital of Traditional Chinese Medicine, Beijing, China

## Abstract

**Objective:**

Triple negative breast cancer (TNBC) is a kind of cancer that endangers the lives of women all over the world in the 21st century. Heat shock protein member 8 (HSPA8) is the chaperone gene of the heat shock protein family. It is involved in many cellular functions. For example, it promotes the circulation between ATP and ADP, participates in protein folding, and can change the vitality of the cell and inhibit its growth. However, the abnormal expression of HSPA8 gene in TNBC and its diagnostic and prognostic significance still need to be further studied.

**Methods:**

First, we used related databases (such as TCGA, GEO, GTEx, ONCOMINE, TIMER2.0, UALCAN, HPA, STRING, CCLE, and Kaplan-Meier plotter databases) to analyze the relationship between HSPA8 and TNBC by bioinformatics. Then, the analysis using only a small part of the experimental work is used to explain our findings. For example, HSPA8 protein expression was evaluated by immunohistochemical method in TNBC tissues. Western blotting experiments were carried out to verify the results. Then, the clinicopathological characteristics of patients with TNBC were analyzed by R software and Cox regression analysis. On the basis, a nomogram is constructed to estimate the 1-, 3-, and 5-year overall survival (OS). The prognostic nomogram performance was calibrated and evaluated by the calibration curve and receiver operating characteristic (ROC) curve.

**Results:**

In the study, we analyzed the three GEO databases (including GSE86945, GSE106977, and GSE102088) and found that HSPA8 is one of the central genes of TNBC. Then, Gene Ontology (GO) and Kyoto Encyclopedia of Genes and Genomes (KEGG) researches indicated that HSPA8 was mainly involved in partner-mediated autophagy, mRNA catabolism, neutrophil activation, immune response, protein targeting, RNA splicing, RNA catabolism, and other biological processes. Next, we used bioinformatics technology to find that the expression level of HSPA8 in breast cancer (BC) and TNBC samples was significantly higher than that in normal breast tissues, which was determined by analyzing hospital patient samples and related experiments. In addition, the expression level of HSPA8 in BC and TNBC samples was significantly correlated with clinical indexes such as TNM stage. The Cox analysis revealed that the expression of HSPA8 in TNBC had significant clinical prognostic value. The results of nomogram and ROC test show that HSPA8 has significant predictive ability in TNBC. The results of immune infiltration of HSPA8 through the TIMER2.0 database showed that there was a significant correlation between HSPA8 and immune cell subsets.

**Conclusions:**

Our results show that the expression of HSPA8 in TNBC has important clinical diagnostic significance and clarify the potential molecular mechanism that promotes the evolution of TNBC. The high expression of HSPA8 may be related with the poor clinical outcome of TNBC. This helps to provide us with a new direction of TNBC targeted therapy.

## 1. Introduction

BC is the most universal cancer that endangers women's lives today [1]. TNBC is a subtype of BC with negative estrogen receptor (ER), progesterone receptor (PR), and human epidermal growth factor receptor–2 (HER–2) [2], accounting for 15–20% of all diagnosed BC, and has the characteristics of strong invasiveness, rapid distant metastasis, and short survival time [3].

Heat shock protein 70 kDa protein (HSP70) is associated with proliferation and metastasis of cancer cells [4]. HSPA8 is one of the important members of HSP70. HSPA8 has the function of molecular chaperone, and it is also a class of structurally expressed proteins that play a significant role in cellular stress response [5]. HSPA8 is overexpressed in a variety of malignant tumor cells, which plays an important role in the occurrence and development of cancer cells [4]. In addition, the absence of HSPA8 can inhibit the growth of solid tumor cells and induce apoptosis and cell cycle arrest [6]. Previous many scholars believe that HSPA8 is involved in tumor molecular chaperone autophagy [7] and participate in the process of BC through molecular chaperone autophagy [8]. Nonetheless, the HSPA8 mRNA abnormal expression in TNBC and its diagnostic and prognostic significance still need to be further studied.

**Figure 1:**
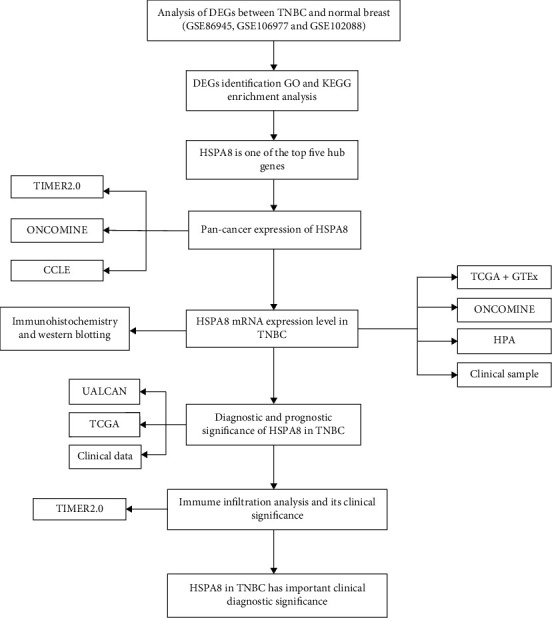
Flowchart related to HSPA8.

Here, the purpose of this study is to explore the expression of HSPA8 in TNBC in detail and in many ways through bioinformatics analysis, clinical sample analysis, immunohistochemistry, and Western blotting. It gives us a deeper understanding of the role of HSPA8 in TNBC. Through the functional analysis, clinical analysis, and immune infiltration analysis of HSPA8 in TNBC, it provides us with a new biomarker of prognosis. The following is the flow chart of this article (Figure 1).

## 2. Materials and Methods

### 2.1. Differentially Expressed Genes

233 cases of TNBC and 114 cases of normal breast specimens from GSE86945, GSE106977, and GSE102088 were analyzed. DESeq2 packet was used in R to identify differentially expressed genes (DEGs) between TNBC samples and normal breast samples [9]. The exclusion criteria are |logFC| > 3 and *P* < 0.01. The volcano map and related heat map of DEGs are drawn by the R software.

### 2.2. GO and KEGG Analyses

The pathways of HSPA8 and DEGs were identified by GO annotation and KEGG. The data are functional enrichment analysis by using clusterProfiler package. [10].

### 2.3. Interaction Analysis

STRING is an online platform for searching known protein-protein interactions and integrating corresponding protein-protein interaction data [11]. STRING was used to evaluate the PPI network of HSPA8 and DEGs of TNBC. The hub genes were screened by CytoHubba's degree algorithm (degree genes≧50 were used as the screening standard).

### 2.4. Data Resource

Retrieve TNBC patient data from the breast invasive carcinoma dataset in TCGA database, comprised of 101 TNBC and 10 paracancerous tissues. The differences between the two groups of samples were analyzed and compared. The TCGA database also provides the TNBC patients corresponding clinical data. For pan-cancer analysis, theTIMER2.0 analyzed the HSPA8 differences between various cancers and adjoin normal specimens.

In addition, 112 cases of TNBC archived in the Affiliated Tumor Hospital of Xinjiang Medical University from January to December 2015 were selected. A total of 112 paraffin specimens of TNBC were collected. All of them were female, and their average age is 54.69 ± 18.12 years. All of them were operated for BC for the first time and were confirmed to be TNBC by pathology after operation. This experiment was approved by the Medical Ethics Committee of our hospital and agreed by these 112 patients.

### 2.5. Comprehensive Evaluation

ONCOMINE is a large tumor gene chip database, covering 65 gene chip datasets, 4700 chips, and 480 million gene expression data [12]. In our research, HSPA8 expression in BC samples and neighbor normal specimen was compared. The screening criteria are as follows: the *P* value is 0.01, the multiple change is 1.5, and the top 10% genes are ranked.

CCLE covers the gene expression of thousands of swollen cell lines from dozens of tissues, so it is a sharp tool for tumor research [13]. The corresponding CCLE data were selected, and the expression of HSPA8 in multiple tumor cell lines was analyzed by the R software (version 4.1.0).

HPA is a free library of immunohistochemical images, which contains dozens of immune expressions of tumors and normal samples [14]. We used the database data to compare the difference of HSPA8 protein in BC and normal breast tissue from the point of view of immunohistochemistry.

UALCAN is a comprehensive network information resource that provides evaluation based on TCGA and MET500 queue data [15]. In the current research, the relationship between HSPA8 and clinicopathological features was analyzed through this platform. *P* < 0.05 was significant.

Kaplan-Meier plotter is a commonly used website for tumor survival analysis [16, 17]. It evaluates the prognostic value of HSPA8 mRNA in BC and TNBC. Survival results included OS and RFS. The optimal cutoff value is determined by the KM plotter algorithm.

### 2.6. Western Blotting

First of all, the cells need to be lysed, and the protein concentration is determined by caprylic acid colorimetry. Then, the protein was separated by sodium dodecyl sulfate-polyacrylamide gel electrophoresis (SDS–PAGE) and transferred to polyvinylidene fluoride membrane. Diluted with 5% bovine serum albumin and Tris buffer saline containing 0.1% Tween 20, the membrane was sealed at room temperature for 2 h and then incubated overnight with anti-HSPA8 primary antibody at 4°C and incubated with secondary antibody for 1.5 h. Protein bands were detected by enhanced chemiluminescence detection kit.

### 2.7. Immunohistochemistry and Result Judgement

Immunohistochemical staining SP method was used to detect the expression of HSPA8 protein in TNBC and benign breast adenosis. The operation steps are carried out in strict accordance with the instructions of the kit. The paraffin blocks of TNBC and benign breast adenosis were cut into 4 *μ*m thick tissue and made into white slices, then dewaxed, hydrated, hot repaired, sealed, and added antibodies (primary antibodies: rabbit polyclonal anti-HSPA8 antibody, 1 : 150, Novus Biological, USA; secondary antibodies: goat anti-rabbit IgG/HRP, 1 : 5000, Invitrogen, USA); DAB kit was stained, dehydrated, transparent, sealed, and observed under a microscope. The expression of HSPA8 was mainly located in the cytoplasm. The immunohistochemical results were interpreted by two pathologists who read the slices double-blindly. And ten visual fields were randomly collected in each case. The percentage of positive cells and staining intensity were observed: (1) staining intensity: no positive staining or cell chromogenic indistinguishability from the surrounding stroma was 0, light yellow was 1, yellow or brownish yellow was 2, and brown was 3 and (2) percentage of positive cells: the number of positive cells < 5% as 0, 5~25% as 1, 25~75% as 2, and >75% as 3. The above two scores were multiplied as the final score of HSPA8 protein expression: 0 as negative, ≥1 as positive, 1 ~ 3 as low expression, and 4 ~ 12 as high expression.

### 2.8. Evaluation of Immune Infiltration

TIMER2.0 used 10,897 cancer samples from TCGA to assess the abundance of immune infiltration [18, 19]. TIMER2.0 gene module was used to study the situation of HSPA8 in different tumors and its relationship with the degree of immune infiltration [20, 21].

### 2.9. Statistical Analysis

Classified measurements are described by counts and percentages, and continuous measurements are represented by mean. Chi-square test was used for classified measurement comparison. The Kaplan-Meier analysis was employed for evaluating patient survival. The purpose of Cox regression was to understand the significance of HSPA8 expression and other clinical parameters in patients with TNBC. The ROC was established by using “PROC package” to analyze the HSPA8 expression significance in diagnosis [22] and using the obtained data to create a nomogram model to analyze the overall survival of patients with TNBC. *P* < 0.05 indicates that the difference is statistically significant.

## 3. Results

### 3.1. Identification of DEGs in Triple Negative Breast Cancer

**Figure 2:**
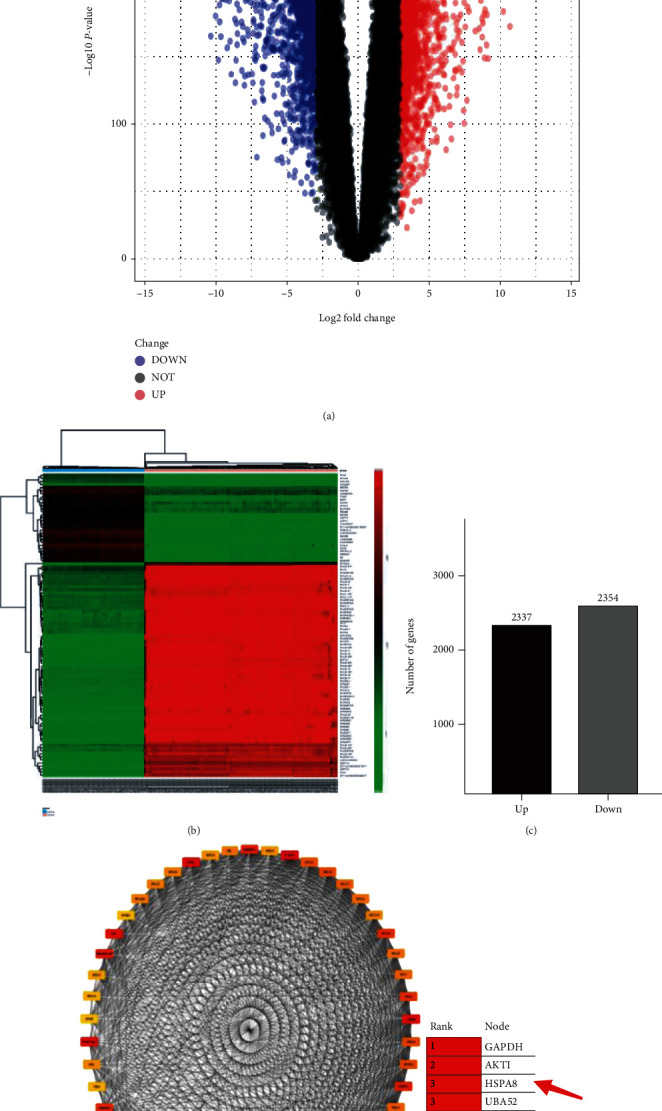
Visualization of differentially expressed genes. (a) Described 4691 DEGs ( |log_2_*FC*| > 3 and *P* < 0.01). (b) The top 100 up- and downregulated DEGs in TNBC and normal breast specimen. (c) The number of DEGs. (d) Top 50 hub genes (by CytoHubba).

Objective to study the abnormal changes of downstream pathway is caused by TNBC differential genes. Three GEO datasets (GSE86945, GSE106977, and GSE102088, containing 233 TNBC and 114 normal breast specimens) were selected. 4691 DEGs were detected in TNBC and normal breast specimens, of which 2337 DEG expressions were upregulated and 2354 DEG expressions were downregulated. These results generate volcanic charts and bar charts, which visually show the result (Figures 2(a) and 2(c)). And the first 100 significantly up- and downregulated DEGs are shown by heat map (Figure 2(b)). The top 1000 down- and the top 1000 upregulated DEGs were input into the STRING database for analysis. The hub genes were screened by degree algorithm in CytoHubba in the Cytoscape software (degree genes≧50 as the screening standard) (Figure 2(d)). Among them, HSPA8 is one of the top five hub genes (Figure 2(d)).

### 3.2. Enrichment Analysis

**Figure 3:**
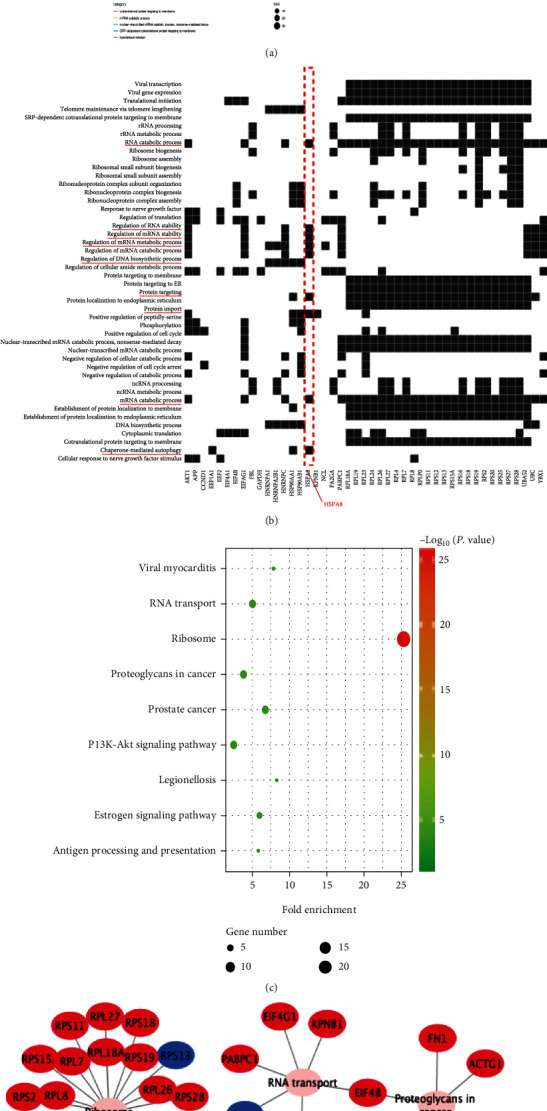
Enrichment analysis. (a) The results of GO analysis of 50 hub genes. (b) The results of GO analysis of HSPA8 gene. (c) KEGG analysis results bubble chart. (d) Metabolic pathway of KEGG.

In order to better demonstrate the possible molecular mechanism of the occurrence and development of TNBC, we used GO and KEGG techniques to evaluate the functions and pathways of 50 hub genes. The results show that biological processes are mainly abundant at the beginning of translation, mRNA catabolism of nuclear transcription, SRP-dependent cotranslation protein targeting membrane, rRNA processing, and so on (Figure 3(a)). Enrichment analysis demonstrated that HSPA8 was involved in partner-mediated autophagy, mRNA catabolism, neutrophil activation, immune response, protein targeting, RNA splicing, RNA catabolism, and other biological processes (Figure 3(b)). The results of KEGG enrichment revealed several main pathways: ribosome, RNA transport, estrogen signal pathway, PI3K–Akt signal pathway, antigen processing and presentation, and proteoglycan in cancer (Figure 3(c)). The genes corresponding to the pathway are listed, such as HSP90AA1 and HSPA8 involved in the estrogen signal pathway (Figure 3(d)). The above results show that these pathways are closely related to the occurrence and development of cancer.

### 3.3. Pan-Cancer Analysis of HSPA8 mRNA

**Figure 4:**
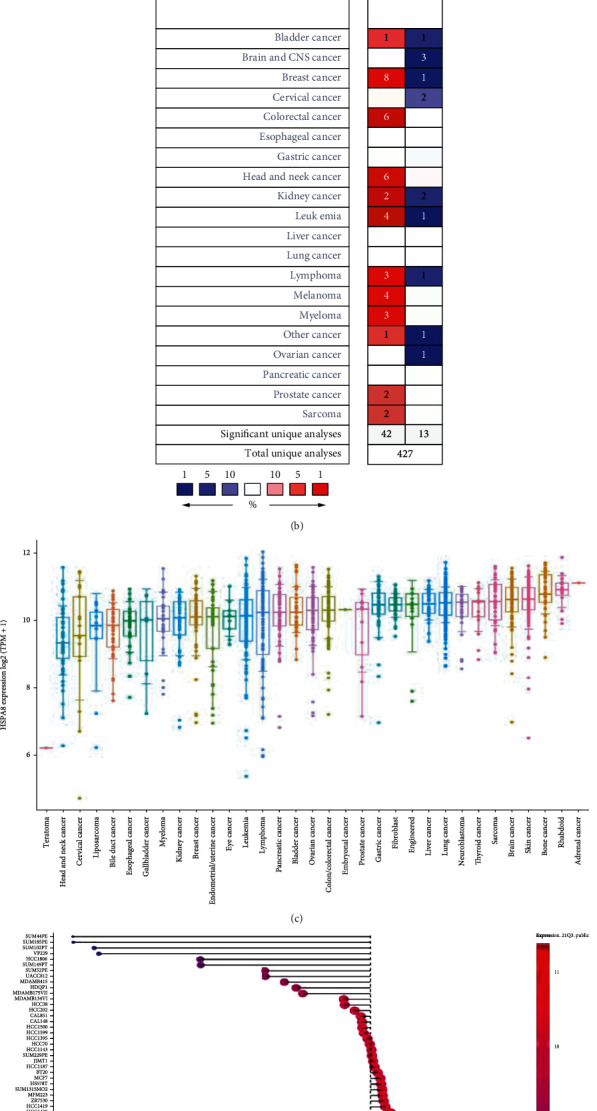
Difference of HSPA8 expression between tumor and paracancerous normal tissue. (a) HSPA8 in 33 kinds of cancer and normal samples adjacent to cancer in the TIMER2.0 datasets. Blue indicates the expression of normal tissue. Red indicates the expression of tumor tissue. (b) The difference of HSPA8 between cancer tissues of different tumor types and adjacent normal samples in the ONCOMINE database (screening criteria *P* < 0.01, 1.5-fold changes (FCs), top 10% gene rank). (c) Expression of HSPA8 in various tumor cell lines in the CCLE database. (d) Expression of HSPA8 in different molecular subtypes of BC cell lines in the CCLE database.

To analyze the expression of HSPA8 in multiple cancers with multiple data from different sources, first, we analyzed the expression of HSPA8 in a variety of tumors and their corresponding normal specimens using TCGA database. In a variety of cancers, including BRCA and head and neck squamous cell carcinoma, the expression of HSPA8 mRNA in tumor tissues was significantly higher than that in normal tissues (Figures 4(a) and 4(b)). Then, the HSPA8 expression level in pan-cancer was evaluated by the ONCOMINE database, which revealed the same result (Figure 4(b)). In addition, through the analysis of the expression of HSPA8 in most tumor cells in the CCLE database, it can be confirmed that the expression of HSPA8 in BC cells is significantly higher than that in other tumor cells (Figure 4(c)). And the HSPA8 expression level in TNBC cell lines (such as HCC1569, SUM159PT, and HCC2157) was higher than that in other BC molecular subtypes (such as UUACC812 and UACC893) (Figure 4(d)).

### 3.4. HSPA8 mRNA Expression in Breast Carcinoma and Triple Negative Breast Cancer

**Figure 5:**
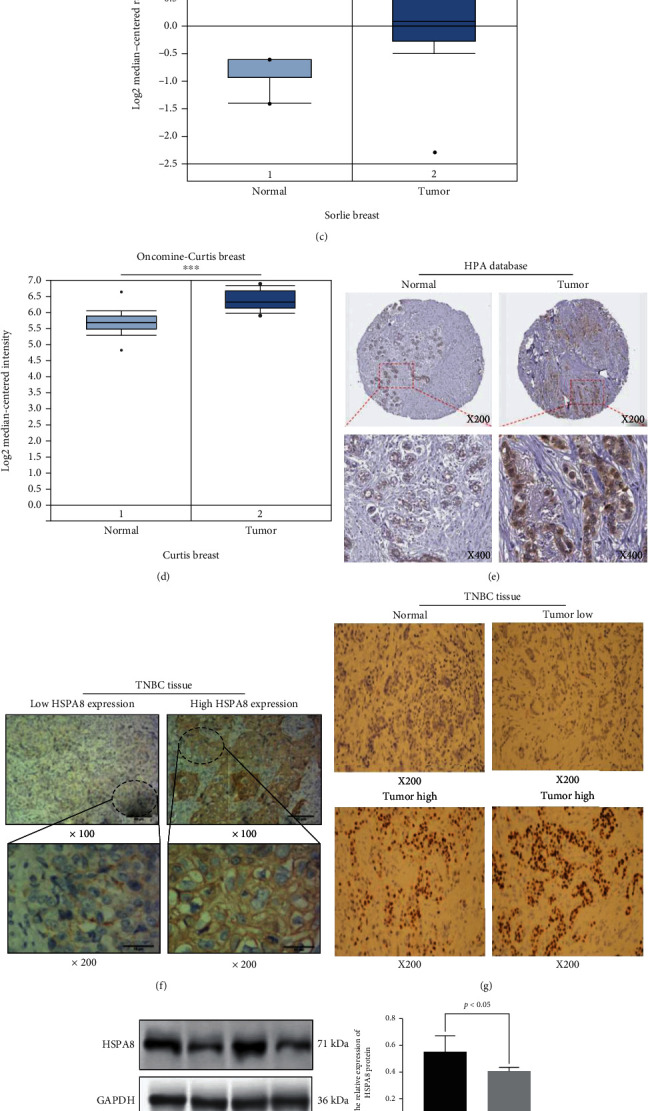
The relative HSPA8 expression in BC and TNBC at the cell and tissue levels. (a, b) Expression of HSPA8 in tumor specimens and normal tissues (using TCGA and GTEx databases). (c, d) Analysis of the expression of HSPA8 in tumor and normal tissues by the ONCOMINE database. (e) The distribution of HSPA8 in BC and normal tissues was shown by the HPA database. (f, g) The results of HSPA8 immunohistochemical detection. (h) Western blotting results of the HSPA8 expression in TNBC and paracancerous tissues. (i) Western blot results of HSPA8 in normal breast and TNBC cell lines.

At present, related studies have shown that HSPA8 is a new cancer biomarker, but the expression of HSPA8 in human TNBC is not clear. Therefore, this study analyzed the transcription of HSPA8 in TNBC tissues and normal breast tissues by TCGA data. It is concluded that there is a significant difference between TNBC and normal breast tissue, and the expression is higher in TNBC (*P* < 0.001) (Figure 5(b)). The same conclusion was obtained in BC (*P* < 0.001) (Figure 5(a)). It is further verified in the ONCOMINE data (*P* < 0.05) (Figures 5(c) and 5(d)). In detail, the datasets of Curtis and Sorlie demonstrate that HSPA8 is upregulated in BC specimens compared with normal specimens, with FCs of 1.579-2.092 (Figures 5(c) and 5(d)). According to the HPA database, high expression of HSPA8 was found in BC tissues (Figure 5(e)). In addition, we obtained the same results by IHC examination of tissue samples from 112 patients with TNBC (Figures 5(f) and 5(g)). The HSPA8 quantitative evaluation in TNBC and paracancerous tissues by Western blot is shown in Figure 5(h) (*P* < 0.05). Finally, the difference of HSPA8 expression between normal breast cell lines and four TNBC cell lines was verified by Western blot quantitative analysis and Western blotting analysis (*P* < 0.05) (Figure 5(i)).

### 3.5. Relationship of HSPA8 and Clinicopathological Parameters of BC and TNBC

**Figure 6:**
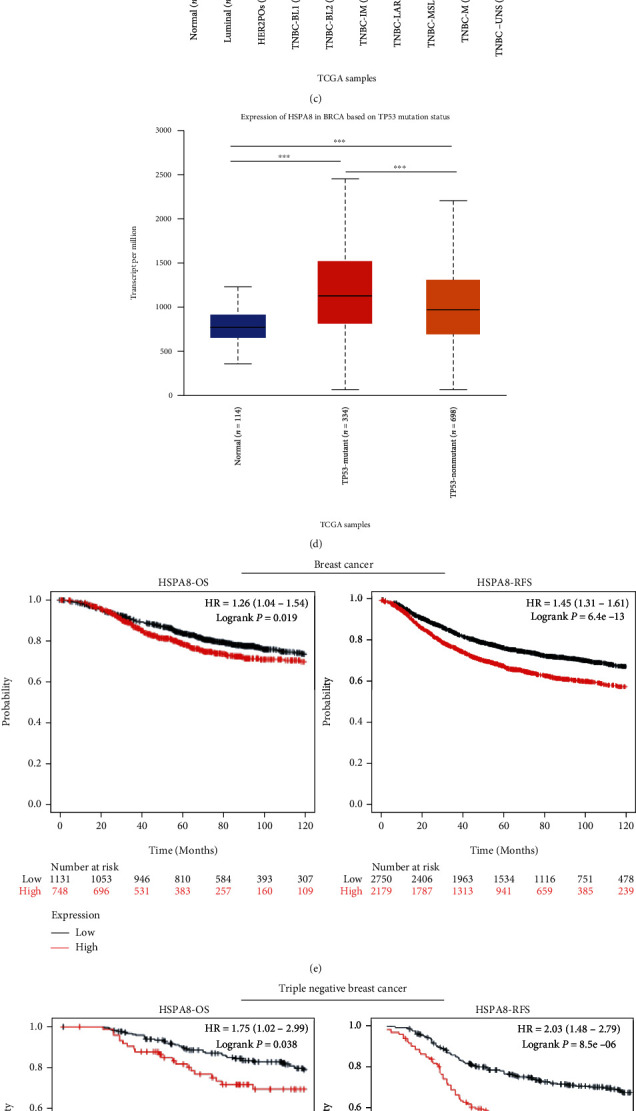
Relationship between HSPA8 and clinical parameters in patients with BC and TNBC and analysis of prognosis. (a–d) Analysis of the relationship between HSPA8 gene expression and individual molecular subtypes, tumor grades, and other clinicopathological parameters in cancer tissues by the UALCAN database. (e, f) The correlation of OS and RFS between HSPA8, BC, and TNBC was analyzed by the Kaplan-Meier plotter database. (g) Relationship between HSPA8 expression and OS time in clinical TNBC patients in our hospital. (h) The relationship between HSPA8 and overall survival in different T stages, N stages, ages, primary stages, and lymph node metastases was analyzed by TCGA database.

In this study, we used the UALCAN database to evaluate the association between HSPA8and the clinicopathologic parameters of BC, including molecular subtype, tumor–stage, pathological grade, and TP53–mutation (Figures 6(a)–6(d)). There were significant differences of HSPA8 found between TNBC group and normal group (Figure 6(a)). And there were significant differences among different TNBC subtypes (*P* < 0.001) (Figure 6(c)). Compared with stage I and stage IV, the expression of HSPA8 was higher in stage II and III. Tumor pathological grade also plays an important role in prognosis. The higher the pathological grade, the higher the expression level of HSPA8 gene in the manifest stage (*P* < 0.05) (Figure 6(b)). The p53 variation reported is closely related to the evolution of cancers [23]. As shown in Figure 6(d), there were differences between the TP53-mutation group and the other two groups (*P* < 0.001). In addition, the expression of HSPA8 in TCGA database was considerably correlated with lymph node metastasis, T stage, and N stage (Figure 6(h)). Moreover, the clinicopathological data of 112 TNBC patients in the Affiliated Tumor Hospital to Xinjiang Medical University were analyzed. Evaluated by logistic statistical method, the HSPA8 expression was related to lots of clinical characteristics of poor prognosis, for example, AJCC stage (OR = 5.846, 95% CI = 2.322–15.641, *P* < 0.001), lymph node metastasis (OR = 6.361, 95% CI = 2.021–28.238, *P* = 0.004), CK5/6 expression (OR = 7.666, 95% CI = 2.439–34.021, *P* = 0.002), and HSPA8 expression (OR = 3.991, 95% CI = 1.601–10.733, *P* < 0.05) (Table 1). In addition, it was found that the survival parameters of patients with TNBC were closely related to the expression of HSPA8 mRNA. The Kaplan-Meier database was used to analyze the survival of patients with BC and TNBC. The results showed that OS (time from onset to death) and RFS (time from initial treatment to recurrence) in the HSPA8 high expression group were slightly lower than those in the HSPA8 low expression group (*P* < 0.001) (Figures 6(e) and 6(f)). The clinicopathological data of 112 patients with TNBC were analyzed. The same conclusion is drawn (Figure 6(g)).

In addition, the relationship between HSPA8 expression and clinicopathological features in cancer tissues of 112 patients with TNBC is shown in Table 2. Using the clinical information of 112 TNBC patients, the Cox regression was used to analyze the prognostic value of HSPA8. This study demonstrated that the high HSPA8 transcriptional was independently associated with the significant shortening of OS (*P* = 0.0035) for TNBC (Table 3). This study shows that the level of HSPA8 transcription is one of the independent prognostic factors affecting the OS of patients with TNBC.

### 3.6. Diagnostic Significance of HSPA8 Expression in TNBC

**Figure 7:**
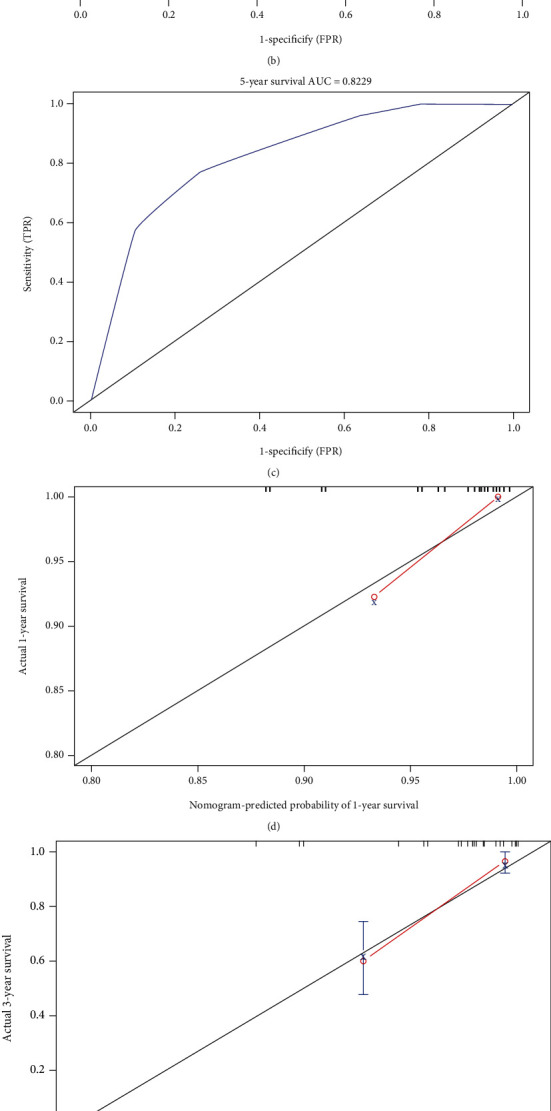
Diagnostic significance of the HSPA8 expression in TNBC. (a–c) The ROC curve of OS in TNBC patients. (d–f) The calibration curve of OS in TNBC patients. (g) Nomogram predicts the probability of OS in TNBC patients.

Next, the HSPA8 diagnostic significance in different clinical features of TNBC patients was evaluated. Using the results of the above multifactor Cox analysis (Table 3), we created a nomogram of the HSPA8 expression and important factors in order to predict the 1-, 3-, and 5-year survival rates of sufferers with TNBC (Figure 7(g)). The higher the nomogram value of OS, the worse the survival and prognosis of the patients. And the *C*-index is 0.801. The calibration curve of the nomogram is close to the 45 degrees, indicating that the nomogram is well calibrated more specifically (Figures 7(d)–7(f)). ROC analysis reveals that the survival line chart could significantly evaluate the 1-, 3-, and 5-year survival rates of sufferers with TNBC. Their AUC values are 0.900, 0.824, and 0.823, respectively (Figures 7(a)–7(c)). These results suggest that the expression of HSPA8 is highly diagnostic in patients with TNBC.

### 3.7. HSPA8 and Immune Infiltrates in TNBC Patients

**Figure 8:**
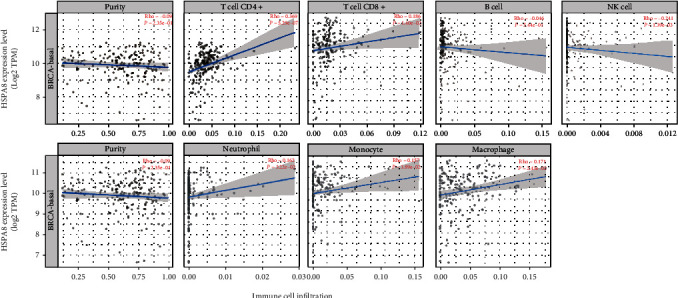
Relationship between HSPA8 mRNA expression and immune cell infiltration (TIMER2.0).

The occurrence and development of tumor are greatly influenced by immune cells in cancer microenvironment [24, 25]. Through the study of the infiltration of diverse immune cells, we demonstrated that there was a positive correlation between the mRNA of HSPA8 and the abundance of immune cells in the microenvironment of TNBC, for example, CD4+, CD8+ T cells, neutrophils, monocytes, and macrophage, but negatively correlated to the abundance of innate immune cells, for example, B cell and NK cell (Figure 8). In addition, the Cox analysis was performed with the TIMER2.0 software to demonstrate the prognostic value of immune cell infiltration and HSPA8 mRNA expression. The results indicated that except for CD8+ T, B cell, NK cell, and neutrophils, the infiltration degree of other 3 kinds of immune cells and the HSPA8 expression were closely related to the clinical characteristics of TNBC (Table 4).

## 4. Discussion

BC is a common malignant tumor in women. In 2020, there were about 19.3 million new cancer patients in the world, of which 11.7% were women with BC, surpassing lung cancer (11.4%) for the first time, becoming the cancer with the largest number of newly diagnosed cancers in the world.. Among them, TNBC has the clinical manifestations of rapid invasion, strong heterogeneity, and short prognosis [26]. Because TNBC lacks effective targets for clinical treatment, it is ineffective to endocrine therapy and is currently the most difficult malignant tumor to treat. At present, the only clinical treatment for patients with TNBC is chemotherapy and radiotherapy, but the risk of recurrence is very high. Therefore, it is very important to study the effective therapeutic targets of TNBC.

HSPA8 plays an important role in the occurrence and development of many organisms, and it is an important chaperone protein. HSPA8 plays an important role in regulating the activity and autophagy of cancer cells. And it has been found that low expression of HSPA8 can inhibit the growth of cancer and stop cell proliferation [6]. In addition, researchers have found that HSPA8 is highly expressed in different cancer cells, such as hepatocellular cancer and endometrial cancer. And it participates in the growth of cancer cells [27, 28] and regulates the autophagy of cancer cells [29]. However, the relationship between HSPA8 gene and TNBC is still unclear. In this research, we discussed the expression of HSPA8 in TNBC comprehensively and systematically. The purpose of this study was to investigate the expression of HSPA8 in TNBC and its relationship with the infiltration level of immune subsets.

First, the HSPA8 expression levels in different kinds of cancers were examined using independent datasets from different platforms (TIMER2.0 and ONCOMINE). HSPA8 was highly expressed in a variety of cancer, such as BRCA, thyroid cancer, cholangiocarcinoma, liver hepatocellular carcinoma, and colon adenocarcinoma. Similarly, high HSPA8 expression was also identified in various tumors cells and TNBC cells by CCLE platform analysis. In summary, these analyses illuminate that HSPA8 may play an important role in the development of tumors. Subsequently, the transcriptional level of HSPA8 in BC and TNBC specimens was significantly higher than that in normal specimens. HSPA8 is highly expressed in hepatocellular carcinoma, prostate cancer and other cancer. [23, 30]. Shan et al. identified that HSPA8 is closely related to endometrial carcinoma by iTRAQ analysis. Previous studies have shown that HSPA8 gene knockout can significantly inhibit the proliferation and promote the apoptosis of endometrial tumor cells [28]. Yang et al. identified that in HBV-related early-stage hepatocellular carcinoma, HSPA8 is thought to be upregulated in tumor tissue and correlated with barren prognosis of patients [31]. In the current study, higher HSPA8 transcription was identified in BC and TNBC samples compared to normal breast samples in various databases. HSPA8 protein expression in BC and TNBC specimens was considerably higher than that in normal breast tissues in TNBC patient samples and HPA platform. In order to further confirm our conclusion, we detected the relative expression of HSPA8 in TNBC tissues, various TNBC, and a normal breast cell line by Western blotting. Finally, we get the same results as bioinformatics analysis.

To further explore the relationship between the expression of HSPA8 and the clinical parameters of TNBC, it was found that the expression of HSPA8 was related to the stage, molecular subtype, TP53 mutation state, and various TNBC molecular subtypes of BC. Logistic regression analysis showed that the expression of HSPA8 was closely associated with lymph node metastasis, T stage, and N stage in patients with TNBC. The Kaplan-Meier test showed that the high expression of HSPA8 indicated that the prognosis of OS and RFS in TNBC patients was poor. Through univariate and multivariate regression analyses, we confirmed that the high expression of HSPA8 is an independent risk factor for the prognosis of TNBC. So far, there is no research report on the construction of TNBC prediction model based on the HSPA8 expression, so we conducted a multi-Cox regression analysis by integrating various clinical parameters from the experimental data, which showed that TNBC tumor stage, lymph node metastasis, CK5/6 expression, and HSPA8 expression were independent prognostic factors, and further created a nomogram to forecast the death risk of individual sufferers and help optimize treatment decisions. The *C*-index index was 0.801. The ROC curve obtained in this study also proved that the expression of HSPA8 is of great value in the diagnosis of TNBC.

In order to study the expression of HSPA8 in TNBC and the downstream pathway of DEGs in TNBC, we identified the DEGs between TNBC and normal breast tissue. The enrichment analysis of GO and KEGG expressed that the above DEGs were primarily involved in ribosome, RNA transport, estrogen signal pathway, PI3K–Akt signal pathway, proteoglycan in tumor, etc. The pathways obtained by the above analysis are closely related to the occurrence and development of malignant tumors.

Some scholars have found that the high expression of HSPA8 is closely related to a variety of carcinogenic activities and signal pathways, such as PI3K–Akt and calcium signaling pathways. For example, targeting PI3K–Akt signal pathway to produce antileukemia effect is to use it to activate upstream oncogenes (such as Flt3–ITD, KIT, and NRAS) [32, 33], and calcium homeostasis disorder plays an important role in the pathogenesis of different kinds of malignant tumors [34]. Studies in patients of HCC with moderate/severe or mild/no depression have found that the high expression of HSPA8 is related to the activation of VEGF/VEGFR2–PI3K–AKT pathway. It is easy to speculate that activating these pathways can lead to poor DFS. Their mechanism is to induce endothelial cell proliferation and migration to promote vascular and tumor growth and to reduce cancer cell apoptosis by inhibiting the expression of BAD and caspase–9 [35] [36]. The changes of PI3K signal pathway are closely related to angiogenesis, tumor proliferation, and inhibition of apoptosis. Tumor gene activation mutations (such as PIK3CA, AKT, and mTOR) are closely related to cancer growth and therapeutic drug resistance. Inactivation mutations of tumor suppressor genes (such as INPP4B and PTEN) are also associated with it [37]. The activation of PI3K is closely related to the occurrence and development of TNBC and chemotherapy resistance [38, 39]. Therefore, we speculate that the high expression of HSPA8 may be related to the activation of PI3K signal pathway in patients with TNBC.

Some scholars have found that tumor growth and survival are accompanied by high activity of chaperone-mediated autophagy (CMA), so there is a close relationship between CMA and tumor [40] [41]. Elevated CMA has been shown to be necessary to maintain enhanced glycolysis to meet rapidly proliferating bioenergy needs [42]. HSPA8 is the key molecular regulator of CMA. A substrate detector will be processed through this special autophagy pathway [43].

Under adverse conditions such as low oxygen content, prolonged hunger [44], oxidative stress [45], or DNA damage, the chaperone Hsc70 can bind to different cochaperones, thus inducing autophagy and protecting tumor cells against cellular death. In addition, studies have shown that upregulation of CMA is necessary for BC cell survival [46]. For example, studies have detected HSPA8 in cancer and paracancerous tissues of patients with the same BC and found higher expression in cancer tissues. And HSPA8 is the key protein of CMA pathway, so it can indirectly indicate that CMA activity is higher in tumor tissue [46]. TNBC is a severe malignant tumor with rapid invasion, early recurrence, and metastasis in breast cancer [47]. Compared with nonmetastatic cell lines, metastatic cell lines have higher levels of basic autophagy, indicating that autophagy can promote invasiveness and may increase tolerance to cellular pressure during metastasis [48]. Some scholars have found that TNBC has a higher level of autophagy, so it is more prone to hypoxia than non-TNBC [49]. Therefore, we speculate that the overexpression of HSPA8 promotes the process of autophagy, which leads to the malignant transformation of TNBC.

At present, more and more evidence supports the hypothesis that immune cell infiltration affects the occurrence and development of tumors, thus affecting the prognosis of patients and the effect of immunotherapy [18]. According to related studies, it has been found that HSPA8 is related to metabolic diseases, cancer, aging, and others [50–54]. And the HSPA8 expression has been found to change in many immune diseases. For example, flow cytometry research has clarified increased expression of HSPA8 on B cell, T cells, and specifically activated T cells in the spleen of MRL/LPR lupus susceptible mice [55, 56]. In-depth analysis of HSPA8 found that it is closely related to the degree of TNBC immune cell infiltration, which is another important finding of this study. The HSPA8 mRNA expression was considerably related to the abundance of CD8+ T, B cells, neutrophils, monocyte, macrophage, and especially CD4+ T cells. Previous researches have illuminated that HSPA8 plays a central role in different key steps of polypeptide antigen presentation by CD4+ T cells, which may regulate the activation of T and B cells [57–64]. This is consistent with the results of our immunoassay for HSPA8. After in-depth analysis, we also found that the expression of HSPA8 was related to the increased infiltration of CD4+ T cells in TNBC. Udono and Srivastava's studies have reported that HSPA8 in cancer cells binds to tumor-specific antigen peptides in order to facilitate host immune system recognition of them [65]. HSPA8 also promotes the transformation of Th cells into Th1 cells by inducing antigen presenting cells to mature, thus directly activating TcR*γδ* T cells and natural killer cells [66].

Natural killer (NK) cells are a kind of cytotoxic lymphocytes and an important part of the innate immune system. Their role in enhancing TNBC antitumor immunity has been widely studied [67]. Some scholars have found that in 75 TNBC patients who received first-line treatment, the baseline circulating tumor cell (CTC) status was positively correlated with peripheral blood NK cells. Baseline CTCs combined with peripheral blood NK count (CTC–NK) can more accurately evaluate progression–free survival in patients with TNBC [68]. NK cells are the main effectors of ADCC. They can effectively kill tumor target cells and play an important role in antibody therapy. In vivo and in vitro studies have shown that L–ICON–CAR–NK cells have a direct killing effect on TNBC cells and mediate L–ICON ADCC to achieve significant effect [69]. Avelumab is a human immunoglobulin anti-PD–L1 monoclonal antibody that triggers ADCC against a group of TNBC cells and enhances natural killer cell-mediated cytotoxicity [70]. Therefore, HSPA8 may directly kill TNBC cells by directly activating NK cells. It is worth noting that macrophage subsets in TNBC tumors tend to coexpress typical M1 and M2 signals, which is the same as the results recently found in human lung and breast cancers [71, 72]. In addition, the Cox proportional hazard model showed that CD4+ T, monocytes, and macrophages were significantly related to clinical severe outcomes in patients with TNBC.

The research reveals the possible mechanism of HSPA8 in the occurrence and development of TNBC and the significance of clinical diagnosis, but there are still some limitations [73]. First, the evaluation of the role of HSPA8 is based on the database and some experiments, which has not been confirmed in vivo, so we need to further explore. Second, although the expression level of HSPA8 and its clinical and prognostic significance have been verified in the clinical specimens of the experiment, and the results are similar to those of the public datasets, it will lead to some errors due to the slight difference in pathological data and the small number of TNBC patients. Finally, the research confirmed that HSPA8 is involved in the regulation of cell cycle and the process of immune infiltration, but its potential molecular mechanism and signal pathway are still unknown. Next, we will study the mechanism of HSPA8 in TNBC.

## 5. Conclusion

In conclusion, the research comprehensively and systematically analyzed the involvement of HSPA8 in the occurrence and development, expression, diagnostic value, survival and prognostic significance, immune infiltration, and possible mechanism of TNBC. Our results provide new markers and treatment targets for patients with TNBC and may provide useful information for accurately predicting the prognosis and treatment of patients with TNBC.

## Figures and Tables

**Table 1:** Relationship between HSPA8 expression and clinical parameters in TNBC by logistic analysis.

Characteristics	Odds ratio (OR)	*P* value
Age (≤60 vs. >60)	0.991 (0.209–3.568)	0.144
Menstruation (no vs. menopause)	0.444 (0.137–1.219)	0.137
AJCC stage (stage II and stage I vs. stage IV and stage III)	5.846 (2.322–15.641)	<0.001
Lymph node metastasis(no vs. yes)	6.361 (2.021–28.238)	0.004
Histologic grade (I and II vs. III)	2.287 (0.874–6.775)	0.108
CK5/6 (no vs. yes)	7.666 (2.439–34.021)	0.002
Distant metastasis (no vs. yes)	—	0.992
HSPA8 expression (low vs. high)	3.991 (1.601–10.733)	0.004

**Table 2:** Relationship between HSPA8 expression and clinicopathological features in BC tissues of patients with TNBC.

Characteristics	All groups	HSPA8		*t*	*P* value
		Low group	High group		
Sample (*n*)	112	63	49		
Age (%)				-0.136	0.892
<35	12 (10.7)	7 (11.1)	5 (10.2)		
35-56	82 (73.2)	46 (73.0)	36 (73.5)		
>56	18 (16.1)	10 (15.9)	8 (16.3)		
Menstruation (%)				0.291	0.590
No menopause	77 (68.8)	42 (66.7)	35(71.4)		
Menopause	35 (31.2)	21 (33.3)	14 (28.6)		
Histologic grade (%)				7.525	0.006
I and II	41 (36.6)	30 (47.6)	11 (22.4)		
III	71 (63.4)	33 (52.4)	38 (77.6)		
AJCC stage (%)				-2.619	0.009
Stage I	18 (16.1)	16 (25.4)	2 (4.1)		
Stage II	56 (50.0)	29 (46.0)	27 (55.1)		
Stage III	32 (28.6)	17 (27.0)	15 (30.6)		
Stage IV	6 (5.4)	1 (1.6)	5 (10.2)		
Lymph node metastasis (%)				10.858	0.001
No	42 (37.5)	32 (50.8)	10 (21.4)		
Yes	70 (62.5)	31 (49.2)	39 (79.6)		
Distant metastasis (%)				9.000	0.003
No	70 (62.5)	47 (74.6)	23 (46.9)		
Yes	42 (37.5)	16 (25.4)	26 (53.1)		
CK5/6 expression (%)				15.368	<0.001
No	46 (41.1)	36 (57.1)	10 (20.4)		
Yes	66 (58.9)	27 (42.9)	39 (79.6)		

**Table 3:** Cox regression analysis of variables for overall survival in TNBC patients.

Characteristics	Univariate analysis		Multivariate analysis	
	Hazard ratio (95% CI)	*P* value	Hazard ratio (95% CI)	*P* value
Age (≤60 vs. >60)	0.89 (0.27–2.97)	0.853	—	—
Menstruation (no vs. menopause)	0.46 (0.17–1.22)	0.119	0.47 (0.18–1.25)	0.129
AJCC stage (stage II and stage I vs. stage IV and stage III)	4.35 (1.94–9.76)	<0.001	4.48 (1.53–13.18)	0.006
Lymph node metastasis (no vs. yes)	5.30 (1.59–17.67)	0.007	4.90 (1.36–47.65)	0.015
Histologic grade (I and II vs. III)	2.13 (0.86–5.32)	0.104	0.55 (0.18–1.73)	0.308
CK5/6 expression (no vs. yes)	6.59 (1.98–21.98)	0.002	9.00 (2.02–40.22)	0.004
Distant metastasis (no vs. yes)	—	0.997	—	—
HSPA8 expression (low vs. high)	3.23 (1.4–7.44)	0.006	2.92 (1.26–6.77)	0.013

**Table 4:** The Cox regression analysis for HSPA8 mRNA expression in TNBC and immune cells (TIMER2.0).

Characteristics	Coef	HR	95% CI_l	95% CI_u	*P* value
CD4+ T	-34.450	0.001	0.001	0.001	0.012 ^∗^
CD8+ T	-0.705	0.494	0.113	2.154	0.348
B cell	-0.434	0.648	0.017	24.094	0.814
Neutrophil	-2.174	0.114	0.004	3.105	0.198
Monocyte	14.379	1.757 × 10^6^	42.334	7.290 × 10^10^	0.008 ^∗∗^
Macrophage	14.103	1.333 × 10^6^	6.679	6.453 × 10^11^	0.035 ^∗^
NK cell	0.133	1.142	0.256	1.885	0.603

^∗^*P* < 0.05,  ^∗∗^*P* < 0.01, and  ^∗∗∗^*P* < 0.001.

## Data Availability

The ONCOMINE, TIMER2.0, UALCAN, HPA, STRING, CCLE, and Kaplan-Meier plotter databases used to support the findings of this study are included within the article. And the HSPA8-Clinical data and TCGA-TNBC-data used to support the findings of this study are included within the supplementary information file(s) (available here).

## Abbreviations

HSPA8: Heat Shock Protein Family A (Hsp70) Member 8
CK5/6: Cytokeratin 5/6
KM: Kaplan-Meier plotter (https://kmplot.com/analysis/)
TCGA: The Cancer Genome Atlas (https://cancergenome.nih.gov/)
GEO: Gene Expression Omnibus
GTEx: Genotype-Tissue Expression
DEGs: Differentially expressed genes
FC: Fold change
PI3K: Phosphoinositide-3 kinase
ADCC: Antibody-dependent cell-mediated cytotoxicity
L-ICON: Tissue factor- (TF-) targeting antibody-like immunoconjugate.

## Supplementary Materials

Supplementary Materials TCGA-TNBC-data. HSPA8-Clinical data.
